# Personal Explanation

**Published:** 1905-04

**Authors:** 


### Foreign Correspondence.

### PERSONAL EXPLANATION.

BERLIN, PARISER PLATZ 7, January 28, 1905.

TO THE EDITOR:

SIR,—Upon the occasion of the suicide of Dr. A. H. Sylvester, of this city, an unjust report has been widely published throughout the United States regarding my withdrawal from his practice, which is unfair to me, and in some instances grossly false. Indeed, although I have taken some trouble to collect the American reports, I have yet to find one correct as to the real facts regarding either his life or his death.

In justice to me, Professor W. D. Miller called a meeting of prominent American dentists here, who, after examining the legal papers, published the inclosed statement in the *German Times* of this city. With their consent I forward same to you, and shall feel very grateful to you, if, in the interest of my good name in the profession, you will give it space in your journal.

As my name has been used in all of the American papers that I have seen, I ask you to kindly insert it in the enclosed.

Very truly yours,

George H. Watson.

"A PROTEST BY AMERICAN DENTISTS IN BERLIN.

"We, the undersigned American Dentists in Berlin, respectfully present the following urgent protest against a notice in the last number of the German Times (Jan. 16th) in which statements are made which are well calculated to injure the name of a member of the American Colony who is most highly esteemed not only as a skilful practitioner of dentistry but as a man of unwavering integrity of character. Having examined into the business relations of this gentleman with the deceased Dr. Sylvester we are absolutely convinced that he not only most conscientiously and scrupulously lived up to the terms of the contract which existed between him and the deceased, but still more that he evinced a charity in his dealings far beyond that which could have been legally exacted and which has evoked our undivided admiration.

"We herewith express our unreserved confidence in the gentleman in question and our strong disapproval of all attempts to make him in any way responsible for a deplorable event over which he had no control.

"Berlin, Jan. 19th, 1905.

Signed:

W. D. Miller. Ferd. Foerster. Charles H. Abbot.

Geo. O. Webster.

George Martin. J. H. Ramsey. E. Lawley-York.

George A. Kennedy. Lee A. Watling.

E. D. Barrows."

